# Association of Blood Donor Sex and Age With Outcomes in Very Low-Birth-Weight Infants Receiving Blood Transfusion

**DOI:** 10.1001/jamanetworkopen.2021.23942

**Published:** 2021-09-03

**Authors:** Ravi M. Patel, Joshua Lukemire, Neeta Shenvi, Connie Arthur, Sean R. Stowell, Martha Sola-Visner, Kirk Easley, John D. Roback, Ying Guo, Cassandra D. Josephson

**Affiliations:** 1 Division of Neonatal-Perinatal Medicine, Department of Pediatrics, Emory University School of Medicine and Children’s Healthcare of Atlanta, Atlanta, Georgia; 2 Department of Biostatistics and Bioinformatics, Rollins School of Public Health, Emory University, Atlanta, Georgia; 3 Department of Pathology and Laboratory Medicine, Emory University School of Medicine, Atlanta, Georgia; 4 Center for Transfusion and Cellular Therapies, Emory University, Atlanta, Georgia; 5 Joint Program in Transfusion Medicine, Brigham and Women’s Hospital and Harvard Medical School, Boston, Massachusetts; 6 Division of Newborn Medicine, Boston Children’s Hospital and Harvard Medical School, Boston, Massachusetts

## Abstract

**Importance:**

There are conflicting data on the association between blood donor characteristics and outcomes among patients receiving transfusions.

**Objective:**

To evaluate the association of blood donor sex and age with mortality or serious morbidity in very low-birth-weight (VLBW) infants receiving blood transfusions.

**Design, Setting, and Participants:**

This is a cohort study using data collected from 3 hospitals in Atlanta, Georgia. VLBW infants (≤1500 g) who received red blood cell (RBC) transfusion from exclusively male or female donors were enrolled from January 2010 to February 2014. Infants received follow-up until 90 days, hospital discharge, transfer to a non–study-affiliated hospital, or death. Data analysis was performed from July 2019 to December 2020.

**Exposures:**

Donor sex and mean donor age.

**Main Outcomes and Measures:**

The primary outcome was a composite outcome of death, necrotizing enterocolitis (Bell stage II or higher), retinopathy of prematurity (stage III or higher), or moderate-to-severe bronchopulmonary dysplasia. Modified Poisson regression, with consideration of covariate interactions, was used to estimate the association between donor sex and age with the primary outcome, with adjustment for the total number of transfusions and birth weight.

**Results:**

In total, 181 infants were evaluated, with a mean (SD) birth weight of 919 (253) g and mean (SD) gestational age of 27.0 (2.2) weeks; 56 infants (31%) received RBC transfusion from exclusively female donors. The mean (SD) donor age was 46.6 (13.7) years. The primary outcome incidence was 21% (12 of 56 infants) among infants receiving RBCs from exclusively female donors, compared with 45% (56 of 125 infants) among those receiving RBCs from exclusively male donors. Significant interactions were detected between female donor and donor age (*P* for interaction = .005) and between female donor and number of transfusions (*P* for interaction < .001). For the typical infant, who received a median (interquartile range) of 2 (1-3) transfusions, RBC transfusion from exclusively female donors, compared with male donors, was associated with a lower risk of the primary outcome (relative risk, 0.29; 95% CI, 0.16-0.54). The protective association between RBC transfusions from female donors, compared with male donors, and the primary outcome increased as the donor age increased, but decreased as the number of transfusions increased.

**Conclusions and Relevance:**

These findings suggest that RBC transfusion from female donors, particularly older female donors, is associated with a lower risk of death or serious morbidity in VLBW infants receiving transfusion. Larger studies confirming these findings and examining potential mechanisms are warranted.

## Key Points

**Question:**

Is the sex or age of a blood donor associated with morbidity or mortality in very low-birth-weight infants receiving blood transfusion?

**Findings:**

In this cohort study of 181 very low-birth-weight infants at 3 centers, infants receiving red blood cell transfusion from female donors had a lower risk of death or serious morbidity compared with those who received transfusion from male donors. The protective association between female donor and adverse outcomes increased with increasing donor age, but diminished with increasing number of blood transfusions.

**Meaning:**

These findings suggest that characteristics of blood donors, such as sex and age, may be associated with recipient outcomes in very-low-birth weight infants receiving blood transfusions.

## Teaser

This cohort study evaluates the association between blood donor sex and age on mortality or serious morbidity in very low-birth-weight (VLBW) infants receiving blood transfusions.

## Introduction

Very low-birth-weight (VLBW) infants commonly receive red blood cell (RBC) transfusions.^1^ Recent randomized clinical trials^2,3^ comparing higher vs lower hemoglobin transfusion thresholds have not reported harm with the use of more liberal hemoglobin transfusion thresholds. Although these trials indicate that more liberal approaches to RBC transfusion are not harmful in preterm infants, they lacked measurement and evaluation of blood donor characteristics.

Several studies^4,5,6^ in critically ill adults have reported associations of female donor sex and younger donor age with an increased risk of mortality. Other studies^7,8^ have reported no association, when controlling for total transfusion exposure. To our knowledge, only 1 prior study^9^ specifically evaluating donor sex has been conducted of VLBW infants and reported that transfusion of RBCs from female donors, compared with male donors, was associated with a higher risk of morbidity and mortality in preterm infants, but this association was no longer significant after accounting for the number of transfusions. In addition, the study^9^ reported an interaction between female donor and number of transfusions with recipient outcome. Another study^10^ reported that 83% of RBC transfusions administered to VLBW infants who went on to develop necrotizing enterocolitis (NEC) were from male donors. To our knowledge, no prior studies have evaluated the effect of donor age on recipient outcomes in preterm infants receiving RBC transfusions.

There is biological plausibility regarding the effects of both donor sex and age on recipient outcomes based on differences in RBC characteristics. RBCs from female donors demonstrate less hemolysis and changes in rigidity during storage compared with those from male donors,^11,12^ and the inflammatory profile of donor blood varies according to sex and age.^13^ In addition, RBCs from female donors and older donors are associated with increased antioxidant capacity, compared with male and younger donors, respectively.^14^ Prior studies in preterm infants have shown that RBC transfusions increase proinflammatory cytokine levels^15,16^ and non–transferrin-bound iron but not oxidant stress,^17^ although none of the aforementioned studies has evaluated the effect of blood donor characteristics. Understanding the effects of donor sex and age may help to understand heterogeneity in the effects of RBC transfusion in preterm infants.

In this cohort study, we evaluated the association of donor sex and age on mortality or serious morbidity in VLBW infants receiving transfusions while controlling for the total number of RBC transfusions. We hypothesized that the characteristics of the RBCs transfused into VLBW infants, specifically the sex and age of the blood donor, would be associated with the risk of death, bronchopulmonary dysplasia (BPD), NEC, or retinopathy of prematurity (ROP).

## Methods

### Setting and Study Population

We performed an analysis of data from the Transfusion-Transmission of Cytomegalovirus study. The study design and results of the study have been previously reported,^18,19^ and this study included an extended cohort of 598 infants, as described elsewhere.^20^ We included VLBW infants born in 3 hospitals in Atlanta, Georgia: Grady Memorial Hospital, Emory University Hospital Midtown, and Northside Hospital. Among the infants enrolled in the parent study from January 2010 to February 2014 (inclusion criteria included birth weight ≤1500 g and postnatal age ≤5 days), we included infants who received an RBC transfusion from exclusively female or exclusively male donors. Infants who received RBC transfusions from both male and female donors were excluded because of the inability to assign a single consistent donor sex exposure. Donor data were linked from information provided by a single blood supplier for all 3 hospitals. However, donor sex information was not recorded on the blood units and was not available to the blood bank personnel as part of routine practice or used to decide allocation of blood units. Standard blood banking practices at the study centers included the use of irradiated, leukocyte reduced, cytomegalovirus seronegative, citrate phosphate dextrose adenine solution, O negative units, regardless of infant blood type, which was not changed during the study period. In addition, the study centers used dedicated single-donor units for each infant to minimize donor exposure for neonatal transfusion. Additional information on donors, such as smoking history, pregnancy history, parity, or medication use, was not available. Parents or guardians provided written informed consent before enrollment of their infant into the Transfusion-Transmission of Cytomegalovirus study. This study was approved by the institutional review board and/or research oversight committees at participating centers and is reported according to the Strengthening the Reporting of Observational Studies in Epidemiology (STROBE) reporting guideline.^21^

### Definitions

The primary outcome was a composite of death, BPD, NEC, or ROP. This composite outcome was selected on the basis of the importance of these outcomes to the risk of long-term neurodevelopmental impairment,^22,23^ the role of oxidant stress and/or inflammation in the pathogenesis of these diseases,^24^ and prior studies^10,25,26,27^ reporting potential associations between RBC transfusion and an increased risk of components of the composite outcome. In addition, a similar composite outcome was used in a prior trial comparing neonatal transfusion thresholds.^28^ We did not evaluate intraventricular hemorrhage,^29^ because we evaluated all RBC transfusions, including those administered after the typical timing of detection of intraventricular hemorrhage. NEC was defined as Bell stage II or higher, with adjudication of staging as described elsewhere.^20^ Moderate-to-severe BPD was defined according to the 2001 consensus definition by the National Institutes of Health,^30^ which was used as the definition at the time the parent study was conducted. ROP was defined as stage III or higher in either eye. Baseline illness severity was assessed using the Score for Neonatal Acute Physiology.^31^ Infants received follow-up until 90 days, hospital discharge, transfer to a non–study-affiliated hospital, or death.

### Conceptual Model

We used a conceptual model to guide modeling of the associations between the study exposures and outcomes (eFigure 1 in the Supplement). The key biasing path between donor sex and age (exposures) and infant characteristics associated with the primary outcome was through transfusion intensity (ie, number of transfusions). Therefore, this variable was adjusted for in multivariable models. In addition, the factor most informative to the risk of adverse outcomes in preterm infants is birth weight,^32,33^ which was included because of its interaction with number of transfusions on the risk of the primary outcome. Interaction between female donor and donor age was tested for in the multivariable model, as well as female donor and number of transfusions, on the basis of a prior study.^9^

### Statistical Analysis

The sample size was fixed according to enrollment into the parent study and study selection criteria. Funding for this secondary study was obtained on July 1, 2019, and initial analyses were completed on December 9, 2020. Missing data were reported, when present, although they were sparse given the quality assurance efforts in the Transfusion-Transmission of Cytomegalovirus study. All statistical tests were 2-sided and conducted at the significance level of *P* < .05. To investigate the relative risks (RRs) of death, BPD, NEC, or ROP, we modeled the primary composite outcome using a modified Poisson regression model with a robust error variance, implemented using the geeglm function in the geepack R package (R statistical software version 3.6.3; R Project for Statistical Computing). The main exposures of interests were female donor and donor age. To reliably and consistently assess the association between donor sex and the primary outcome, we performed the analysis using data from infants who exclusively received RBC transfusion from only female donors or only male donors. Donor age for an infant was measured as the mean donor age of all RBC transfusions an infant received, which was then centered at the mean across infants. Birth weight, mean donor age of all RBC transfusions an infant received, and the number of RBC transfusions were specified as continuous variables. Interaction terms to determine whether the association between donor sex and the primary outcome depended on donor age or number of transfusions were retained in the model only if they were significant (*P* < .05). In the presence of significant interactions, RRs were presented graphically at different levels of the risk factors in addition to reporting of the regression model parameter estimates.

Next, we conducted sensitivity analyses to evaluate the alternative assumptions that additional biasing paths between donor characteristics and the primary outcome exist through gestational age, duration of RBC storage or storage after irradiation, baseline illness severity, exposure to multiple donors, or center, that may not have been fully accounted for by adjustment for the number of transfusions. In addition, we performed a sensitivity analysis of a subset of infants who received only a single RBC transfusion, similar to a prior study evaluating donor sex.^7^ This model included donor age and birth weight, but no interaction terms, because there could be no plausible interaction with the number of transfusions and the smaller sample size. Finally, we evaluated the association between donor sex and each individual component of the composite outcome. Because of the relatively small number of events for some of the components, we analyzed each outcome separately with exact logistic models with the main effects of donor sex, donor age, total number of transfusions, and birth weight and reported odds ratio estimates from the exact logistic regression.^34^

## Results

Among an available population of 598 infants, we included 343 infants who received RBC transfusion. We had covariate and RBC donor information available for 324 infants (95%) who received 1650 RBC transfusions, of which 655 (40%) were from female donors. Among these 324 infants, 143 received RBC transfusions from both male and female donors and were excluded, allowing for analysis of 181 infants who received 499 RBC transfusions. Of these 499 RBC transfusions, 136 (27%) were from female donors.

The mean (SD) birth weight of the cohort was 919 (253) g, and the mean (SD) gestational age was 27.0 (2.2) weeks. Fifty-six (31%) of the 181 included infants received RBCs from exclusively female donors, and the remainder received RBCs from exclusively male donors. The average of the mean (SD) donor age was 46.6 (13.7) years (range, 17-74 years). Female donors were 2 years younger than male donors (mean [SD], 45.0 [13.9] vs 47.3 [13.6] years) (eFigure 2 in the Supplement). The median (interquartile range [IQR]) number of RBC transfusions was 2 (1-3) transfusions, which was similar for infants receiving RBC transfusion from only female donors or only male donors (eTable 1 in the Supplement). The median (IQR) number of transfusions was 3 (2-5.5) transfusions among infants with the primary outcome and 1 (1-2) transfusion for infants without the primary outcome (Table 1). Most infants received 1 to 3 transfusions from a single donor (eFigure 3 in the Supplement). By contrast, the median (IQR) number of transfusions among infants who were excluded who received RBCs from both male and female donors was 7 (4-11) transfusions (eTable 1 in the Supplement).

**Table 1.** Characteristics of Infants Receiving Blood Transfusions and Blood Donors

Characteristic	Mean (SD)^a^	
	Infants without primary outcome (n = 113)	Infants with primary outcome (n = 68)
Infant characteristics		
Birth weight, g	979 (241)	819 (241)
Gestational age, wk	27.4 (2.2)	26.4 (2.2)
Sex, No. (%)		
Female	51 (45)	32 (47)
Male	62 (55)	36 (53)
Score for Neonatal Acute Physiology	11.2 (4.5)	13.0 (4.2)
Transfusions, No.	1.8 (1.2)	4.3 (4.1)
Transfusions, median (IQR), No.	1 (1-2)	3 (2-5.5)
Blood donor characteristics		
Sex, No. (%)		
Female	44 (39)	12 (18)
Male	69 (61)	56 (82)
Age, y^b^	46.5 (14.2)	46.8 (13.0)
Transfusion from multiple donors, No. (%)	26 (23)	31 (46)
RBC storage age, d^b^	9.5 (4.3)	10.4 (4.0)
RBC storage age after irradiation, d^b^	1.8 (2.7)	1.8 (2.7)

Abbreviations: IQR, interquartile range; RBC, red blood cell.

^a^ Complete data were available for all characteristics.

^b^ Mean for all infants was calculated using the mean for all RBC transfusions for an individual infant.

The primary outcome occurred in 68 infants (38%), with 44 infants with moderate-to-severe BPD, 17 infants who died, 13 infants with NEC, and 4 infants with severe ROP. The primary outcome incidence was 21% (12 of 56 infants) among infants receiving RBCs from exclusively female donors, compared with 45% (56 of 125 infants) among infants receiving RBCs from exclusively male donors. Similarly, among infants who developed the primary outcome, 18% (12 of 68 infants) received RBC transfusions from exclusively female donors, compared with 39% (44 of 113 infants) among those who did not develop the primary outcome. Additional blood donor characteristics are listed in Table 1.

**Figure 1.**
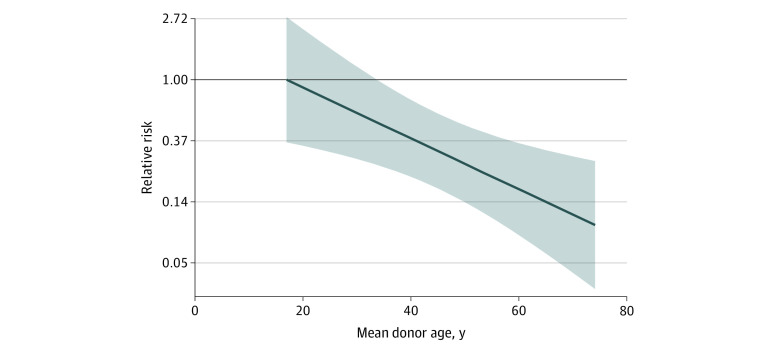
Association of Blood Donor Sex and Age With Primary Outcome Relative risk of the primary outcome (dark blue line) and corresponding 95% CIs (shaded area) comparing infants receiving red blood cells from only female donors vs only male donors is shown by mean donor ages. Values less than 1 indicate a protective female donor association. All relative risks were calculated at a total of 2 transfusions, which was the median transfusion intensity for the cohort.

**Figure 2.**
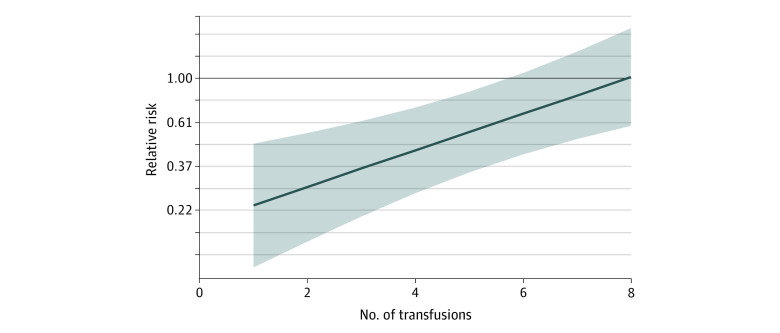
Association of Blood Donor Sex and Number of Transfusions With Primary Outcome Relative risk of the primary outcome (dark blue line) and corresponding 95% CIs (shaded area) of the primary outcome comparing infants receiving red blood cells from only female donors vs only male donors is shown by the number of transfusions. Values less than 1 indicate a protective female donor association. All relative risks were calculated at the mean centered donor age value in order to remove the interaction between centered donor age and female donors.

In multivariable analyses, we detected significant interactions between female donor and mean donor age (*P* for interaction = .005), female donor and number of transfusions (*P* for interaction < .001), and number of transfusions and birth weight (*P* for interaction = .006) (eTable 2 in the Supplement). For the typical infant, who received a median of 2 transfusions, receipt of RBC transfusions from exclusively female donors, compared with male donors, was associated with a lower risk of the primary outcome (RR, 0.29; 95% CI, 0.16-0.54) (Table 2). As donor age increased, the protective association between female donor sex and the primary outcome increased and moved further away from the null (eg, RR, 1.0) (Figure 1 and Table 2), but diminished as the number of transfusions increased (Figure 2). For example, the model-based RR of the primary outcome was 0.24 (95% CI, 0.12-0.49) for infants receiving 1 RBC transfusion and 1.03 (95% CI, 0.60-1.78) for infants receiving 8 RBC transfusions. The lowest RRs of the primary outcome comparing female vs male donors were observed among infants who received fewer RBC transfusions from older donors (eFigure 4 in the Supplement). Among a subset of 76 infants who received only 1 RBC transfusion, receipt of RBC transfusion from a female donor was associated with a lower risk of the primary outcome (RR, 0.15; 95% CI, 0.02-0.99) after adjustment for birth weight and donor age.

**Table 2.** Estimates of Association of Exposures with Primary Outcome

Variable	Relative risk of primary outcome (95% CI)^a^
Female vs male donor (overall)^b^	0.29 (0.16-0.54)
By different mean donor ages^c^	
38.6 y (25th percentile)	0.41 (0.21-0.77)
48.6 y (50th percentile)	0.27 (0.14-0.50)
57.6 y (75th percentile)	0.18 (0.09-0.38)
Birth weight per 100 g increase^d^	0.89 (0.82-0.98)

^a^ Multivariable model was used to derive estimates and included the following main effects: female donor sex, mean donor age (centered at the mean for all transfusions an infant received), total number of transfusions, and birth weight (in grams). In addition, the following interaction terms were included: total number of transfusions by female donor sex, total number of transfusions by birth weight, and mean donor age (centered) by female donor sex. Parameter estimates are reported in eTable 1 in the Supplement.

^b^ Refers to estimate at the median number of transfusions of 2 per infant and mean donor age.

^c^ Refers to estimate of female vs male donor sex at the median number of transfusions of 2 per infant and the listed mean donor age.

^d^ Refers to estimate at the median number of transfusions of 2 per infant and same donor sex.

**Figure 3.**
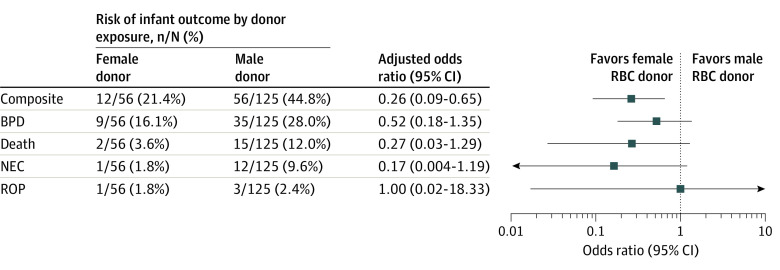
Risk of Individual Components of the Composite Outcome by Donor Exposure Estimates from exact logistic models for each outcome separately with female red blood cell (RBC) donor as the parameter of interest and total transfusions, birth weight, and donor age as nuisance parameters. Birth weight was binarized at 1000 g, and mean donor age was binarized at the mean for all infants. BPD indicates bronchopulmonary dysplasia; NEC, necrotizing enterocolitis; and ROP, retinopathy of prematurity.

Expanded models that included gestational age (model 2), storage age of RBCs (model 3), storage age following irradiation of RBCs (model 4), multiple donor exposure and Score for Neonatal Acute Physiology (model 5), and center (model 6) yielded similar estimates as the primary analysis (eTable 2 in the Supplement). Of these additional covariates, only center was associated with the primary outcome. Analyses evaluating individual components of the composite outcomes showed estimates that were generally consistent with the association between receipt of RBC transfusions from exclusively female donors and the composite outcome, although estimation was imprecise (Figure 3).

## Discussion

In this cohort study, we found that among VLBW infants receiving RBC transfusions from exclusively female or exclusively male donors, those who received RBC transfusions from exclusively female donors, vs exclusively male donors, had a lower risk of death or serious morbidity (21% vs 45%). In addition, the protective association between RBC transfusions from exclusively female donors, compared with male donors, and a lower risk of death or serious morbidity increased with increasing donor age. These findings suggest that blood donor sex and age may be associated with recipient outcomes in VLBW infants receiving transfusions. Because many VLBW infants receive transfusions repeatedly and often receive multiple transfusions from a single donor because of the common practice of dedicated donor units, we believe this is an ideal population to assess the potential impact of blood donor characteristics on transfusion recipient outcomes.

Our results differ from those of the single-center study by Murphy et al,^9^ which did not find association between donor sex and morbidity or mortality in preterm infants after adjusting for the number of RBC transfusions. This may be the result of the inclusion of infants who received both male and female donor RBCs as an exposure group, or other differences in modeling approaches, study populations, or transfusion practices. Of note, infants who received transfusion of RBCs from both male and female donors and were excluded in our study received a median of 7 transfusions, compared with a median of 2 transfusions for infants who were included. This suggests that infants who received transfusions from both male and female donors may have had greater illness severity, as also suggested by the high incidence of adverse outcomes in this group. We believe this is the most likely explanation for the differences in findings between our study and that of Murphy et al.^9^ Both our study and the one by Murphy et al^9^ found an interaction between female donor sex and the number of transfusions. In our study, the protective association between female donor sex and a lower risk of death or serious morbidity diminished with an increasing number of RBC transfusions. This finding may be the result of the higher incidence of the composite outcome as the number of RBC transfusions increased, because most infants receiving high numbers of transfusions either die or survive with morbidity.^35^

The association between donor characteristics and outcomes has been conflicting in adult studies, with several demonstrating an association^4,5,6^ and others no association^7,8^ after controlling for total transfusion exposure. These studies suggest the potential for confounding by transfusion intensity because those patients who receive more RBC transfusions may be more likely to receive units with less common donor characteristics. Because of this, our conceptual model focused on this key biasing path, and all models controlled for the total number of transfusions. In addition, the results of our primary analyses were consistent in a sensitivity analysis in a subset of infants who received only 1 RBC transfusion. To test alternative assumptions, we also conducted 5 sensitivity analyses to evaluate the possibility that additional biasing paths between donor characteristics and outcome exist that are not accounted for by adjustment for transfusion intensity. The estimates from these analyses, which included adjustment for gestational age, RBC storage age, RBC storage age after irradiation, baseline illness severity (using Score for Neonatal Acute Physiology), exposure to single or multiple donors, and center, were consistent with the primary model results with minimal to no change in estimates for the 2 exposures of interest. In addition, the age and sex of blood donors are not considered in the allocation of RBC units by blood banks to specific infants, so we did not consider confounding by indication to be a likely source of bias. Furthermore, routine practice at study centers was to provide only type O blood, and storage duration was not determined according to donor characteristics. Considering this along with the similar duration of storage age of transfused RBCs among infants with and without the primary outcome, and the lack of change in model estimates after adjusting for storage age, it is unlikely that storage age confounded the associations between the study exposures and primary outcome.

In adults, female donor sex has been associated with worse outcomes, possibly because of immunological changes that occur during pregnancy.^4^ By contrast, in our study we found female donor sex to be associated with a lower risk of death or serious morbidity. This may be due to different mechanisms, as immune-mediated transfusion reactions are uncommon in preterm infants. In contrast, transfusion of adult RBCs into neonates^36^ and RBC deformability may potentially influence blood flow,^37^ and there is better deformability of RBCs among female donors compared with male donors.^11,12^ In addition, RBC transfusions in preterm infants may be inflammatory,^15,16^ potentially through release of factors from hemolysis, and the inflammatory profile of RBCs is reduced in female and older donors.^5,13^ However, these potential mechanisms are speculative, and additional studies are needed to further evaluate the potential biological mechanisms that may explain the associations observed in this study.

The results from recent multicenter randomized clinical trials^2,3^ comparing higher vs lower hemoglobin transfusion thresholds in preterm infants have found no differences in short-term or long-term adverse outcomes. These findings suggest that more liberal RBC transfusion may not be harmful in preterm infants. In contrast, the present study focused on the RRs of donor characteristics for a fixed number of transfusions that a VLBW infant may receive. Therefore, donor sex could potentially be associated with recipient outcomes, even with RBC transfusion approaches using higher or lower thresholds. Our findings support the importance of measurement and evaluation of blood donor characteristics in future studies examining the relative benefits and harms of RBC transfusion.

### Limitations

Our study has limitations. We were unable to determine additional donor characteristics. Smoking,^38^ pregnancy,^4^ or medication use^39^ affect donor RBC characteristics and may explain the associations observed between donor sex and age with the study outcome, although we considered these as explanatory rather than confounding factors. We approached our modeling strategy with a conceptual framework that may not have accounted for all potential biasing paths. However, expanded models in our sensitivity analyses that tested alternative assumptions were consistent with the primary analyses. In addition, the use of a composite outcome may be limited by divergent associations within individual components of the composite outcome. However, we found estimates of association with each component of the composite that were directionally consistent with the overall primary analysis, with the exception of ROP. In addition, there is the potential that outcomes such as death or NEC may have occurred after the follow-up period, although on the basis of our prior study,^40^ few deaths occur after 90 postnatal days and most initial cases of NEC occur before 60 postnatal days.^20^ Furthermore, we believe our findings should be validated in larger cohort studies, and, if confirmed, evaluated in clinical trials as is currently being done for RBC transfusion for adults.^41^

## Conclusions

In conclusion, in this cohort study, RBC transfusion from female donors, particularly older female donors, was associated with a lower risk of death or serious morbidity in VLBW infants receiving transfusions. Larger studies confirming our findings and examining potential mechanisms are warranted.

## Notes

Supplement. **eFigure 1.** Conceptual Model **eFigure 2.** Distribution of Donor Age Stratified by Sex **eFigure 3.** Number of Transfusions by Number of Donors **eFigure 4.** Relative Risk of the Primary Outcome by Donor Age and Number of Transfusions **eTable 1.** Incidence of Outcomes by Donor Sex **eTable 2.** Parameter Estimates From Primary and Expanded Models
