# Supplementary material for: Association of Blood Donor Sex and Age With Outcomes in Very Low-Birth-Weight Infants Receiving Blood Transfusion
Source: JAMA Netw Open. 2021 Sep 3;4(9):e2123942. doi: 10.1001/jamanetworkopen.2021.23942 (PMC8417762; doi:10.1001/jamanetworkopen.2021.23942)
Supplement: Supplement. — eFigure 1. Conceptual Model eFigure 2. Distribution of Donor Age Stratified by Sex eFigure 3. Number of Transfusions by Number of Donors eFigure 4. Relative Risk of the Primary Outcome by Donor Age and Number of Transfusions eTable 1. Incidence of Outcomes by Donor Sex eTable 2. Parameter Estimates From Primary and Expanded Models [file jamanetwopen-e2123942-s001.pdf]

Supplemental Online Content

Patel RM, Lukemire J, Shenvi N, et al. Association of blood donor sex and age with outcomes in very low-birth-weight infants receiving blood transfusion. *JAMA Netw Open*. 2021;4(9):e2123942. doi:10.1001/jamanetworkopen.2021.23942

eFigure 1. Conceptual Model
eFigure 2. Distribution of Donor Age Stratified by Sex
eFigure 3. Number of Transfusions by Number of Donors
eFigure 4. Relative Risk of the Primary Outcome by Donor Age and Number of Transfusions
eTable 1. Incidence of Outcomes by Donor Sex
eTable 2. Parameter Estimates From Primary and Expanded Models

This supplemental material has been provided by the authors to give readers additional information about their work.

© 2021 Patel RM et al. *JAMA Network Open.*

eFigure 1. Conceptual Model

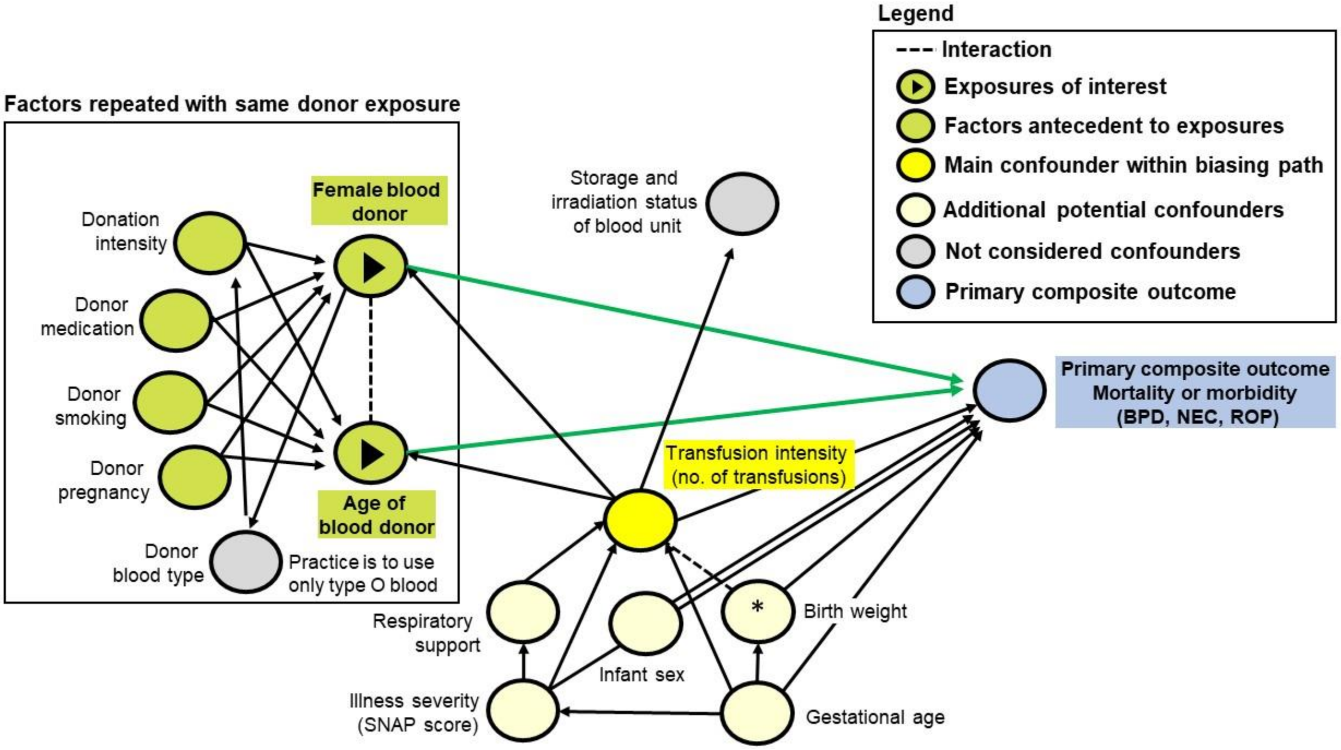

Legend: Figure depicts a conceptual model of associations between exposures of interest (green circles with black triangles), factors antecedent to exposures of interest (green circles), primary outcome (blue circle), main confounder (yellow circle), additional potential confounders within biasing path of number of transfusions (light yellow) and covariates that were not considered confounders (gray circles). Green arrows indicate the associations of interest between the study exposures and outcome. Dotted lines indicate potential interaction. Because red blood cell transfusions are not allocated based on donor age or sex, the main biasing path between the exposure and outcome is transfusion intensity, as a greater number of transfusions (or potentially a greater rate or volume of transfusions) may influence utilization of blood bank inventory of red blood cells from more common vs. less common donor types (blood units from female donors are less common than male donors). Because storage and irradiation of red blood cells are not based on donor characteristics, this was not considered a potential confounder for the primary analysis. As routine practice is to use type O blood for neonatal red blood cell transfusion, donor blood type was not considered a potential confounder. Figure edited after being initially generated using DAGitty (Johannes Textor, Benito van der Zander, Mark K. Gilthorpe, Maciej Liskiewicz, George T.H. Ellison. Robust causal inference using directed acyclic graphs: the R package 'dagitty'. International Journal of Epidemiology 45(6):1887-1894, 2016).
Abbreviations: BPD, bronchopulmonary dysplasia; NEC, necrotizing enterocolitis; ROP, retinopathy of prematurity; SNAP, Score for Neonatal Acute Physiology.

© 2021 Patel RM et al. *JAMA Network Open.*

eFigure 2. Distribution of Donor Age Stratified by Sex

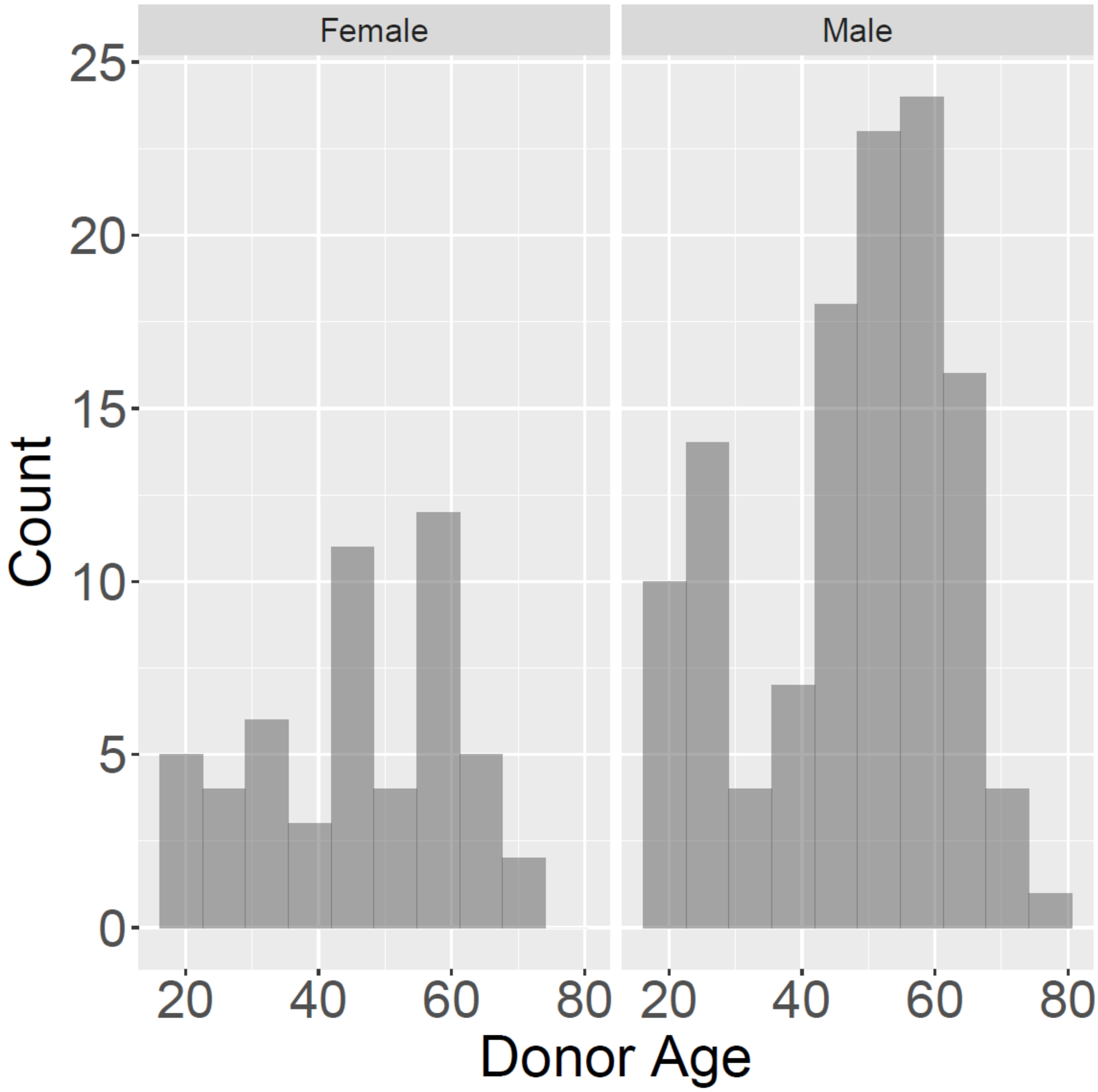

Legend: This figure shows the distribution of donor age among male and female donors. The mean age of female donors was 45.0 years (standard deviation 13.9). The mean age of male donors was 47.3 years (standard deviation 13.6).

© 2021 Patel RM et al. *JAMA Network Open.*

eFigure 3. Number of Transfusions by Number of Donors

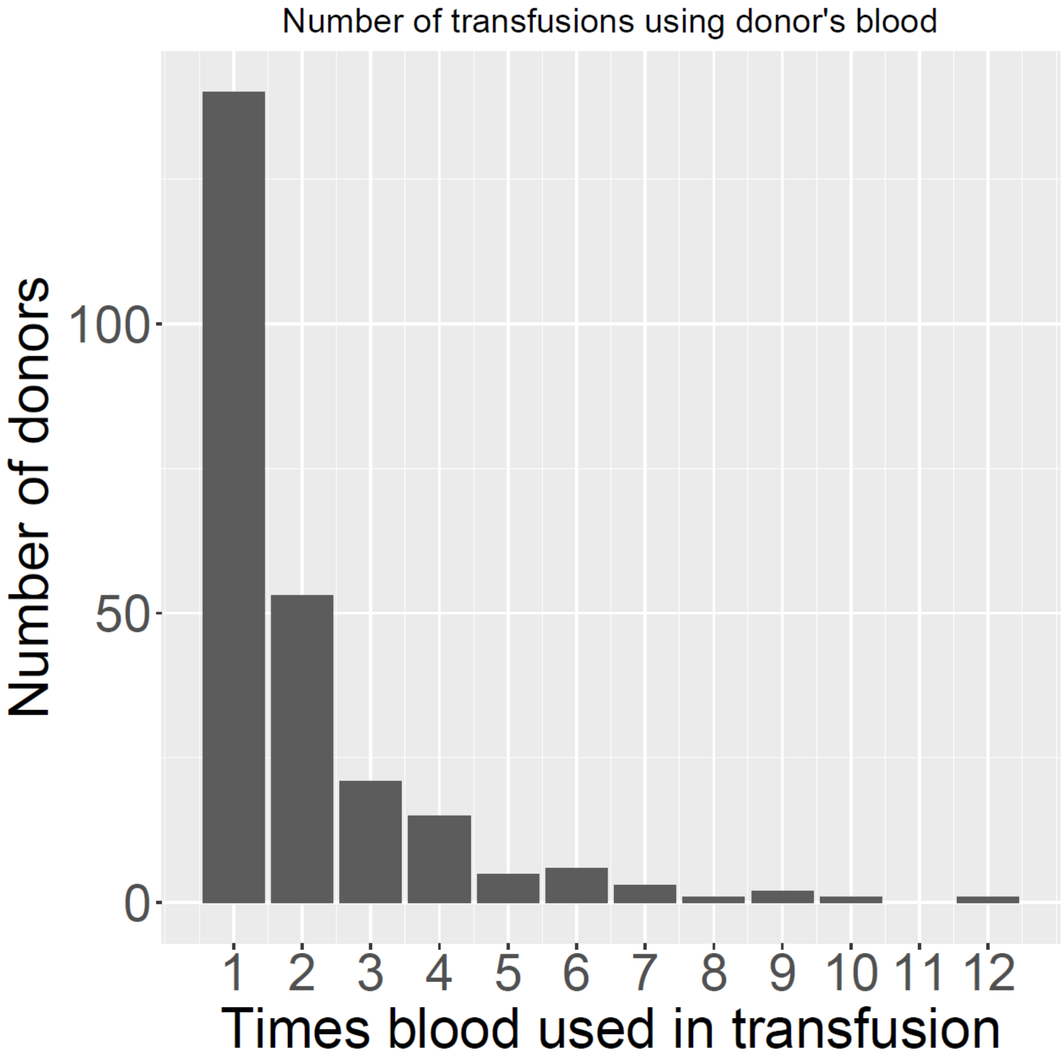

Legend: This figure shows multiple donor exposures on the x-axis (number of times a donor's blood was used for red cell transfusion), by the number of donors on the y-axis. Most infants received 1-3 red cell transfusions from a single donor.

© 2021 Patel RM et al. *JAMA Network Open.*

eFigure 4. Relative Risk of the Primary Outcome by Donor Age and Number of Transfusions

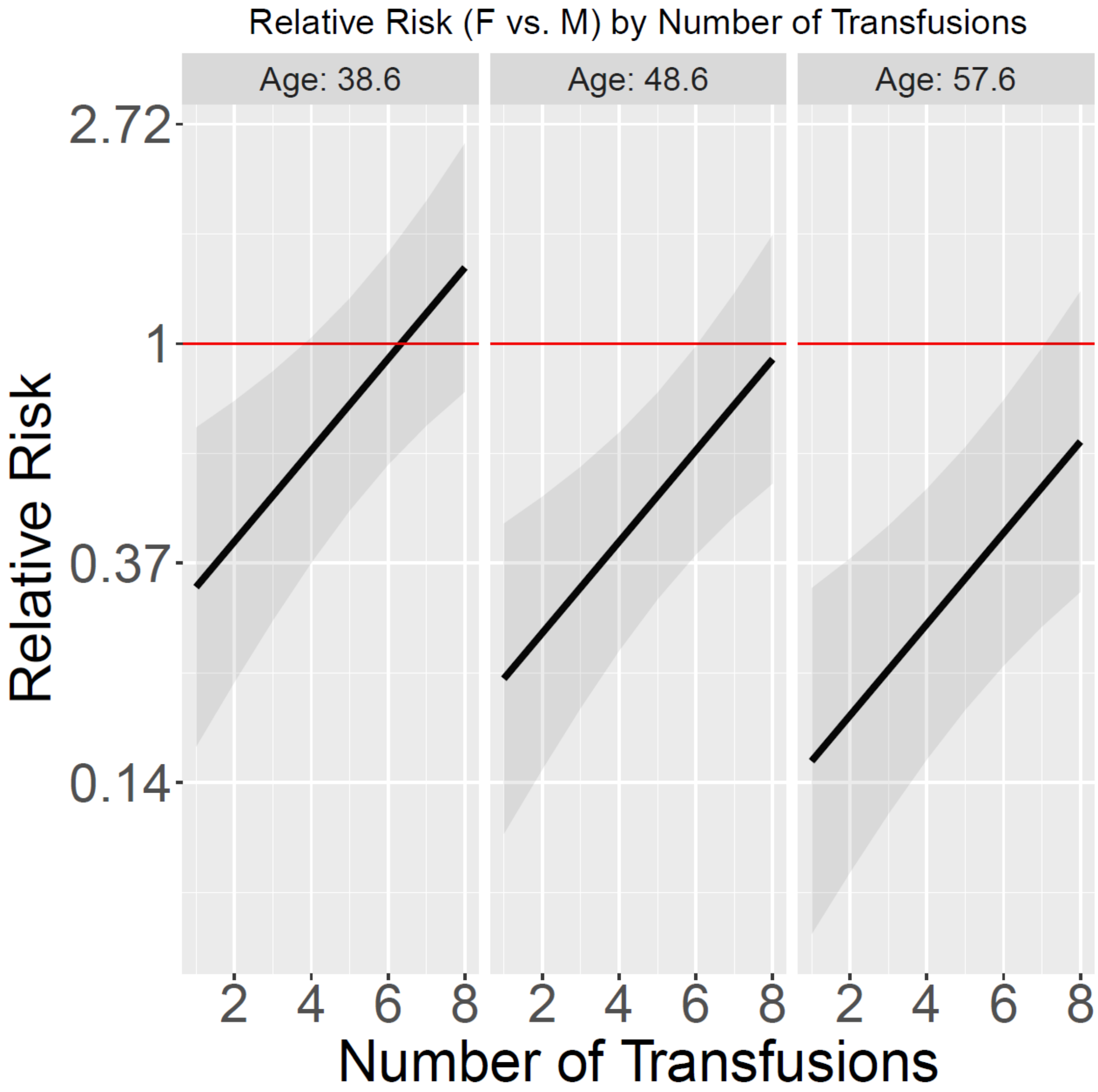

Legend: This figure reports relative risk estimates (black lines), with corresponding 95% confident intervals (gray shaded areas) to account for the interaction between donor age and donor sex as well as the number of transfusions and donor sex. The relative risks of the primary outcome are shown, comparing infants who received red cell transfusion from exclusively female donors (F) to exclusively male donors (M), by the number of transfusions (x-axis) and the ages of donors at the 25th, 50th and 75th percentile of mean donor ages (top headings and vertical panels). Values less than 1 (red line) indicate a protective association between transfusion of RBCs from female donors and the risk of the primary composite adverse outcome.

© 2021 Patel RM et al. *JAMA Network Open.*

eTable 1. Incidence of Outcomes by Donor Sex

Outcome	Infants with only female donor RBC transfusion	Infants with only male donor RBC transfusion	Infants with both female and male donor RBC transfusion[a]
Composite	12/56 (21.4%)	56/125 (44.8%)	102/143 (71.3%)
Death	2/56 (3.6%)	15/125 (12.0%)	14/143 (9.8%)
BPD	9/56 (16.1%)	35/125 (28.0%)	82/143 (57.3%)
NEC	1/56 (1.8%)	12/125 (9.6%)	30/143 (21.0%)
ROP	1/56 (1.8%)	3/125 (2.4%)	16/143 (11.2%)
Number of transfusions, median (25th, 75th percentiles)	2 (1, 3)	2 (1, 3)	7 (4, 11)

Data are reported as n/N (%), unless otherwise noted.
Abbreviations: RBC, red blood cell; BPD, bronchopulmonary dysplasia; NEC, necrotizing enterocolitis; ROP, retinopathy of prematurity.

[a] These infants were excluded from the primary analyses, because a single consistent donor sex could not be assigned.

© 2021 Patel RM et al. *JAMA Network Open.*

eTable 2. Parameter Estimates From Primary and Expanded Models

Variable	Model 1: Primary model	Model 2: Addition of gestational age	Model 3: Addition of storage age of RBCs	Model 4: Addition of storage age of RBCs post-irradiation	Model 5: Addition of multiple donor exposure and illness severity	Model 6: Addition of center
Female donor sex	-1.651 ± 0.406, <0.0001	-1.668 ± 0.403, <0.0001	-1.649 ± 0.406, <0.0001	-1.618 ± 0.406, <0.0001	-1.780 ± 0.436, <0.0001	-1.717 ± 0.401, <0.0001
Mean donor age (Centered)	0.007 ± 0.007, 0.29	0.007 ± 0.007, 0.31	0.007 ± 0.007, 0.32	0.007 ± 0.007, 0.25	0.007 ± 0.007, 0.34	0.005 ± 0.007, 0.49
Total number of transfusions	-0.151 ± 0.074, 0.04	-0.165 ± 0.081, 0.04	-0.152 ± 0.074, 0.04	-0.150 ± 0.074, 0.04	0.036 ± 0.019, 0.06	-0.144 ± 0.075, 0.05
Birth weight (per 1 gram)	-0.002 ± 0.001, 0.002	-0.002 ± 0.001, 0.003	-0.002 ± 0.001, 0.003	-0.002 ± 0.001, 0.0008	-0.001 ± 0.001, 0.009	-0.002 ± 0.001, 0.004
Total number of transfusions x Female donor sex	0.208 ± 0.062, 0.0008	0.212 ± 0.060, 0.0004	0.207 ± 0.062, 0.0007	0.201 ± 0.063, 0.002	0.177 ± 0.068, 0.009	0.225 ± 0.059, 0.0001
Total number of transfusions x Birth weight	0.0003 ± 0.0001, 0.006	0.0003 ± 0.0001, 0.007	0.0003 ± 0.0001, 0.006	0.0003 ± 0.0001, 0.007	[a]	0.0003 ± 0.0001, 0.007
Mean donor age (Centered) x Female donor sex	-0.042 ± 0.015, 0.005	-0.042 ± 0.015, 0.005	-0.041 ± 0.015, 0.006	-0.039 ± 0.016, 0.01	-0.045 ± 0.018, 0.01	-0.045 ± 0.015, 0.004
Gestational age (per 1 week)		0.036 ± 0.049, 0.46				
Mean RBC storage age (per 1 day)			-0.003 ± 0.023, 0.88			
Mean RBC storage post-irradiation (per 1 day)				0.030 ± 0.033, 0.37		
Multiple donor exposure (yes vs. no)					0.219 ± 0.204, 0.28	
SNAP score (per 1 unit)					0.003 ± 0.021, 0.88	
Center A						-0.651 ± 0.312, 0.04
Center B						-0.389 ± 0.221, 0.08

Estimates shown are model coefficients (β) ± SE, *P*, from multivariable models that include all variables with parameter estimates provided in the corresponding column, fitted to the composite primary outcome. Empty cells indicate that variable was not included in the multivariable model. Sample sizes are the same for each model (n=181) because there was no missing data for any variable.
Abbreviations: RBC, red blood cell; SNAP, Score for Neonatal Acute Physiology.

[a] Not included to avoid overfitting model.

© 2021 Patel RM et al. *JAMA Network Open.*
